# Antibody-Drug Conjugates: The New Frontier of Chemotherapy

**DOI:** 10.3390/ijms21155510

**Published:** 2020-07-31

**Authors:** Sara Ponziani, Giulia Di Vittorio, Giuseppina Pitari, Anna Maria Cimini, Matteo Ardini, Roberta Gentile, Stefano Iacobelli, Gianluca Sala, Emily Capone, David J. Flavell, Rodolfo Ippoliti, Francesco Giansanti

**Affiliations:** 1 Department of Life, Health and Environmental Sciences, University of L’Aquila, I-67100 L’Aquila, Italy; sara.ponziani@guest.univaq.it (S.P.); giuseppina.pitari@univaq.it (G.P.); annamaria.cimini@univaq.it (A.M.C.); matteo.ardini@univaq.it (M.A.); rodolfo.ippoliti@univaq.it (R.I.); 2 MediaPharma SrL, I-66013 Chieti, Italy; g.divittorio@mediapharma.it (G.D.V.); r.gentile@mediapharma.it (R.G.); s.iacobelli@mediapharma.it (S.I.); g.sala@unich.it (G.S.); 3 Department of Medical, Oral and Biotechnological Sciences, University of Chieti-Pescara, I-66100 Chieti, Italy; caponemily@gmail.com; 4 The Simon Flavell Leukaemia Research Laboratory, Southampton General Hospital, Southampton SO16 6YD, UK; davidf@leukaemiabusters.org.uk

**Keywords:** Mabs, Antibody-Drug Conjugate, cancer therapy, drug targeting, payload, cross-linking

## Abstract

In recent years, antibody-drug conjugates (ADCs) have become promising antitumor agents to be used as one of the tools in personalized cancer medicine. ADCs are comprised of a drug with cytotoxic activity cross-linked to a monoclonal antibody, targeting antigens expressed at higher levels on tumor cells than on normal cells. By providing a selective targeting mechanism for cytotoxic drugs, ADCs improve the therapeutic index in clinical practice. In this review, the chemistry of ADC linker conjugation together with strategies adopted to improve antibody tolerability (by reducing antigenicity) are examined, with particular attention to ADCs approved by the regulatory agencies (the U.S. Food and Drug Administration (FDA) and the European Medicines Agency (EMA)) for treating cancer patients. Recent developments in engineering Immunoglobulin (Ig) genes and antibody humanization have greatly reduced some of the problems of the first generation of ADCs, beset by problems, such as random coupling of the payload and immunogenicity of the antibody. ADC development and clinical use is a fast, evolving area, and will likely prove an important modality for the treatment of cancer in the near future.

## 1. Introduction

The twentieth century has been characterized by basic and applied research leading to the discovery and use of an increasing number of cytotoxic chemotherapeutic compounds with the ability to rapidly kill dividing cancer cells in preference to non-dividing healthy cells. The well-known drawback of chemotherapy is due to the fact that these drugs, in addition to damaging cancer cells, also damage healthy tissues; thus, causing side effects, sometimes with serious consequences.

The challenge is, therefore, to search for drug delivery systems that achieve high cytotoxic efficacy against cancer cells, but with limited systemic toxicity. Antibody-drug conjugates (ADCs) offer the promise of achieving this objective and increase the therapeutic index significantly.

The approach to targeted chemotherapy comes from Paul Ehrlich’s concept of the “magic bullet” formulated at the beginning of the twentieth century [1]. The principle of this concept, to avoid side effects, drugs must be guided and released into the tumor sites through association with ligands that are overexpressed or selectively expressed in the tumor. Ehrlich’s proposal has been translated into practical applications for therapy due to the development of monoclonal antibodies in the mid-70s, combining the selectivity of recognition to the power of chemotherapeutic drugs [2]. To become a pharmacologically active drug, monoclonal antibodies can be linked to either a radioisotope (giving rise to Antibody radioimmunoconjugates, RAC), to a highly potent cytotoxic drug (antibody-drug conjugates, ADCs) or protein toxins (producing immunotoxins) [3,4].

The production of ADCs face several vital issues, such as the target cell selection, the nature of antigen, structure and stability of the antibody, the linker chemistry, and finally the cytotoxic payload.

One of the first problems encountered in the use of antibodies was the fact that murine antibodies are foreign proteins recognized as non-self by the human immune system that responds by producing human anti-mouse antibodies (HAMA). HAMAs can have toxic effects due to immune-complex formation in the patient and, thus, prevent further administration. With the technology of recombinant DNA, Phage display, and transgenic mice, it is now possible to create of completely human antibodies that are not immunogenic and greatly ameliorate such toxicities.

Chemotherapeutic drugs include antimetabolites (methotrexate, 6-mercaptopurine, 5-fluorouracile, cytarabine, gemcitabine, etc.), molecules interfering with microtubule polymerization (vinca alkaloids, taxanes), and molecules inducing damages on DNA (anthracyclines, nitrogen mustards). The most recent generation of chemotherapeutic molecules include both DNA damaging/alkylating agents (i.e., duocarmycin from Medarex/Bristol Mayer Squibb, Syntarge, calicheamicin from Wyeth/Pfizer, indolino-benzodiazepine from Immunogen), and molecules interfering with microtubule structure (i.e., maytansinoids, from immunogen, auristatin derivatives from Seattle Genetics). These compounds can kill cells with extremely high potency so that severe side effects greatly limit the administrable dose as a free drug. These compounds are therefore considered as ideal payload components of ADCs with high therapeutic index [5].

The conjugation strategy and chemistry chosen to represent a key factor for the success of ADCs, the homogeneity of ADC molecules being one of the main challenges in ADC design [2]. In deciding in which chemical conjugation process to use, it is necessary to develop a strategy that allows the reaction of those residues placed on the surface of the antibody through a chemical reactive group present on the linker. These strategies, depending on the type of residue (mainly amino groups of lysines or sulfhydryl groups of cysteines) that can lead to the production of mixed species whose Drug-Antibody Ratio (DARs) is variable. When the DAR is poorly controlled, this phenomenon can reduce the efficacy of the ADCs and furthermore increase aggregation possibility, the overall rate of clearance and release of the payload systemically at an early stage [6], although higher DAR values are beneficial for the overall potency. To improve the technology, focusing on obtaining homogeneous ADCs with a high therapeutic index, site-specific conjugation technologies have now been developed [7].

## 2. Basic Characteristics of the Conjugate

**Figure 1:**
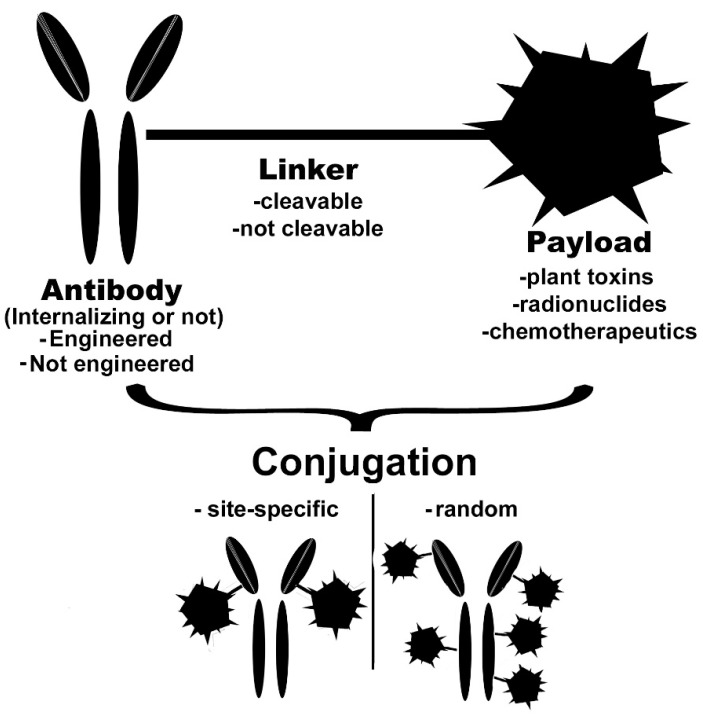
Schematic representation of various types of antibody-drug conjugates (ADCs) and their components.

An ADC is composed of three different components (Figure 1): a monoclonal antibody, the payload, and the linker that joins the first two components. Different types of conjugation chemistry exist: as in the most common, linkage is obtained through lysine (ε-amine-group, -NH_2_ in the deprotonated form) or cysteine (sulfhydryl-group, -SH). However, other conjugation strategies may also be pursued (see below). Whatever the conjugation strategy, it is vital that this does not affect the integrity and functionality of the antibody.

### 2.1. Monoclonal Antibody

In the development of ADCs for cancer treatment, the choice of the antigen and, consequently, selection of the appropriate antibody plays a key role.

The antibody is chosen based on the molecular target recognition, with the highest affinity and selectivity for the target. Ideally, it should recognize an overexpressed target only at the tumor site to avoid delivering the pharmacological load inappropriately to non-target sites. For example, the (human epidermal growth factor receptor 2) (HER2) receptor is more than 100 times overexpressed in tumor tissues in comparison to the equivalent normal non-cancerous tissue [8].

The antigen against which the antibody is directed on the cancer cell should be present in high copy number (>10^5^/cell.) [9]. So far, several antigens have been reported overexpressed in cancer tissues that can be exploited as targets for ADCs [10]. The antigen must be recognized and bound by the antibody with a reasonable affinty (Kd ≤ 10 nM) to ensure rapid uptake in the target cell [11].

In the first generation of ADCs, in many cases murine antibodies being recognized as foreign proteins generated a strong immune response with the production of anti-human antibodies that potentially reduced their therapeutic efficacy. This problem has been partially solved through the use of genetic engineering in second-generation ADCs, utilizing a mouse-human chimeric antibody format. The “humanized” chimeric antibody contains the mouse light and heavy chain variable regions that are linked to human constant regions. The chimeric ADCs showed promising results in cancer treatment but sometimes the problem of decreased efficiency and human anti-chimeric response were still present.

To overcome this problem, many efforts have been made to design a humanized monoclonal antibody, which contain only murine complementary determining regions (CDRs) regions combined with the human variable region [8] or fully human antibodies [12].

Usually, the antibodies used to construct ADCs are of the IgG1 class (Immunoglobulin G Subclass 1) (~150 kDa), but since antibodies in ADCs exploit the Fab region to recognize the antigen present at the end of light chains, only this region is essential to the antibody to carry out its function as a specific carrier. Therefore, in some cases, smaller antibody formats (i.e., antibody fragments that maintain the binding affinity for the receptor) have been used to create ADCs. These fragments can be obtained by IgG cleavage following papain digestion or recombinant production to produce Fabs and scFvs [13].

Selected antibodies and their derived ADCs can be directed against antigens that may or may not induce internalization through receptor-mediated endocytosis (RME), and by this criterion, ADCs can be classified as internalizing or non-internalizing.

#### 2.1.1. Internalizing ADCs

Internalizing ADCs exploit RME to be internalized by target cells. In this case, the antibody performs a fundamental role as it favors the internalization of the target antigen receptor, which represents a crucial step for most ADCs to be effective. Although, as in the case of the anti-HER3 antibody EV20, the binding to the receptor and the internalization of receptor/antibody complex can alone induce cell death and inhibition of tumor growth [14,15,16].

Following internalization, the ADC can follow different endocytic routes that crucially may have profound effects on their cytotoxic efficacy. Clathrin-mediated and caveolae-mediated endocytosis (CME) in which the receptor mediates endocytosis and, alternatively, clathrin-caveolin-independent endocytosis, where the receptor does not mediate endocytosis [17]. The most common route to reach the cell cytoplasm, adopted by various ADCs, is CME, which is target antigen dependent. Molecules, such as epsin, dynamin, adaptor protein 2 (AP2), and phosphatidylinositol (4,5) bis-phosphate (PIP2) may increase accumulation of ADCs on the surface of cellular membrane [18] and assist the internalization of the ADC into the endo-lysosomal vesicle compartment.

**Figure 2:**
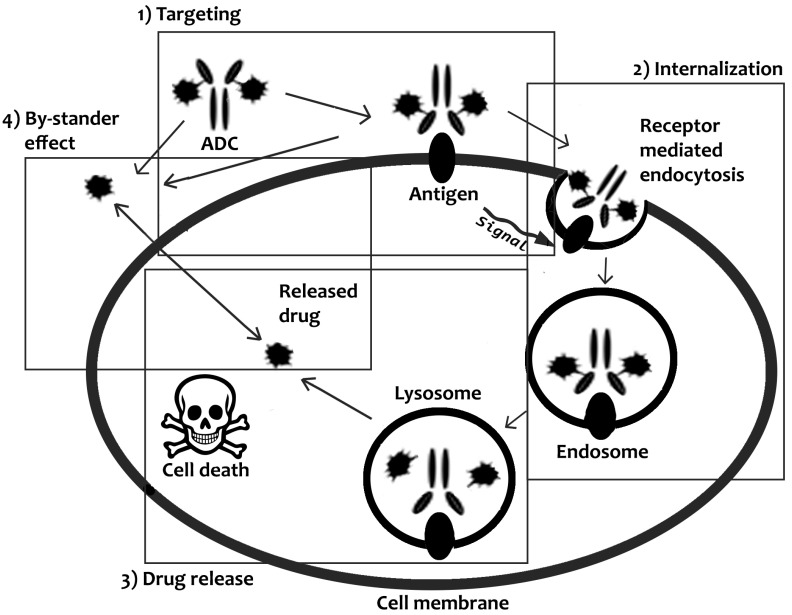
Schematic illustration of the mechanism of drug delivery and release mediated by ADCs.

Early endosomes form just below the membrane surface and usually endo-lysosomal vesicles containing ADCs progress to form late endosomes, whose lumens are acidic and may lead to the dissociation of antibodies from their receptors thus playing a vital role in recycling of antigen back to the membrane surface and subsequently lead to fusion of the late endosomal vesicle with lysosomes. The resulting pH decrease may also result in degradation of the ADC due to the numerous proteolytic enzymes present in the acidic lysosomal compartment with subsequent release of the drug payload. [19] (Figure 2).

Release of the drug within endolysosomal vesicles then results in the passive transport of drug payload into the cytosol where it can exert its pharmacological effect, killing the cancer cells via a molecule specific mechanism [20,21].

#### 2.1.2. Non-Internalizing ADCs

The main pharmacological action of ADCs constructed with non-internalizing antibodies, relies on the cytotoxic payload exerting a bystander effect upon reaching the target tumor site. In this instance, once the ADC reaches the tumor site, proteolytic enzymes, or the reducing conditions in the tumor extracellular environment, act to liberate the drug payload, which facilitate the entry of drugs into the cells, by diffusion, pinocytosis, or other mechanisms. Once the released drugs start kill cancer cells, they release additional reducing agents or proteases, which in turn catalyze further release of drugs (Figure 2). This type of conjugates may also allow a by-stander effect on non-target cancer cells that are near the main target tumor mass, due to diffusion of the released drug into neighboring tumor cells of the drug [22].

It has been reported that an ADC directed against the alternatively spliced extracellular domain A of fibronectin induces a potent anticancer effect following the release of its payload after tumor cell death in the extracellular milieu. This allows the diffusion of the cytotoxic drug also into neighboring cells, and amplification of the process determined by a further release of reducing agents (e.g., cysteine, glutathione) [23].

### 2.2. Linkers

The linker component of the ADC, through which the covalent chemical bond between the drug and the antibody is created, should be chosen rationally, based on the mechanism of action of the antibody (whether internalizing or not) and limit potential chemical modifications to the drug in order to avoid loss of cytotoxicity. One of the main aims for the effective systemic delivery of an ADC is that the drug is released only at the target site; the linker, thus, must be stable enough in a biological environment (i.e., blood circulation) to avoid unwanted release of the pharmacological molecule.

**Figure 3:**
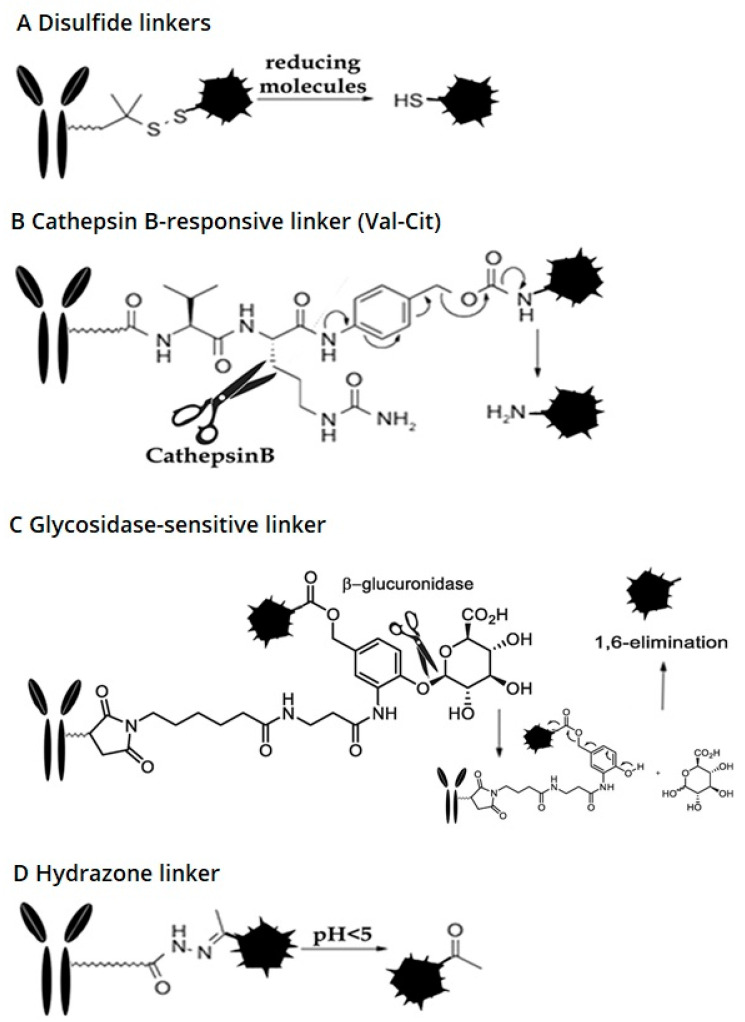
Available cleavable linkers in ADC (**A**) Disulfide linker, cleaved by reducing agents; (**B**) cathepsin B responsive linker, cleaved by Cathepsin B; (**C**) glycosidase-sensitive linker, cleaved by gluconidase; (**D**) hydrazone linker, cleaved by acidic environment.

There are two types of linkers available: cleavable and non-cleavable (Figure 3). The former can be used either in the design of either an internalizing or not internalizing ADC, because the release of the payload is required to take place in either the extracellular tumor environment, or within the lysosome or cytosol. This is possible because the extracellular environment of the tumor is highly reducing due to the presence of glutathione, which allows the release of payloads linked to the antibody via thiolic bonds. It also allows payload release via the degradation of peptide bonds in the presence of proteases such as Cathepsin B, whose overexpression in cancer drives its normal lysosomal localization towards extracellular secretion [24]. A cleavable linker, therefore, exploits differential conditions of reducing power or enzymatic degradation that can be present either outside or inside the target cell. Due to the chemical reactions needed to release the payload, the site of conjugation on the antibody is crucial to induce both stability in the plasma and availability to reduction or degradation on/into the target cell [25,26]. Non-cleavable linker-based ADC must, however, be internalizing, because to release their cytotoxic payload, the antibody component needs to be degraded by lysosomal or cytoplasmic proteases [27]. Furthermore, drugs linked to such linkers usually cannot exert a by-stander effect because upon degradation of the antibody by cellular proteases, they are released as fragments of antibody peptides that have a poor ability to permeate the cells. This type of non-cleavable linker has a higher efficiency for the treatment of tumors that express an antigen at high levels (to achieve a good clinical response and tumor regression, 99% of targeted cancer cells must be eliminated) or for hematological tumors [28,29].

#### 2.2.1. Cleavable Linkers

Figure 3 above summarizes the most commonly used cleavable linkers that are described in detail in the sections that follow.

##### Disulfide Linkers

This type of linker is glutathione-sensitive. The disulfides are stable at physiological pH, in the systemic blood stream, but they are vulnerable to nucleophilic attack by thiols. Human serum albumin (HSA) represents the main thiol in plasma, being its concentration as high as >400 mM. Notwithstanding this high concentration, HSA fails to break the disulfide bond of ADC because its residue containing free thiol (Cys34) is found near a cleft in the molecule that is not significantly exposed to the solvent [30]. Conversely, disulfide-linked drugs resist reductive cleavage in the circulation because the glutathione (GSH) concentration in the blood (5 µmol/L) is lower than in the cytoplasm (1–10 mmol/L) allowing GSH thiol groups to be very effective in the cell cytoplasm also due to its well exposed position and its small size [31]. This difference in reductive potential between plasma and cytosol allows for the selective release of the intracellular payload of the ADCs. In addition, cancer cells cause oxidative stress that generates high GSH levels. Low glutathione levels in healthy tissues therefore discriminate release of the payload, also allowing the selective release of payload in close proximity to the tumor. ADCs with disulfide linkers are often associated with maytansinoid payloads, which were originally developed by Immunogen in 1992 [32]. To increase the stability of the bond, methyl groups may be added to surround disulfides in the linker structure [33], such as in the case of *N-*succinimidyl-4-(2-pyridyldithio)pentanoate (SPP) containing a single methyl, or *N-*succinimidyl-4-(2-pyridyldithio)butanoate (SPDB) containing two methyl groups.

Some ADC designs use a direct disulfide bond between the drug and the antibody. In this variety of ADC, the release of the drug is completely dependent on a strongly reducing tumor microenvironment [34]. Recently, ADCs with a direct disulfide bond between engineered cysteine residues and the thiols of maytansinoids payloads have been investigated [30]. By protecting disulfides reduction through antibody hindrance, these ADCs have good in vivo stability in mouse plasma. The results demonstrate that the DM3 payload is more stable than the DM1, given that only 10% of disulfide bonds are cleaved in plasma, a property that confers increased in vivo therapeutic activity in a murine model [35]. The structure of the whole antibody thus represents a protective environment significantly reducing the reductive release of the payload in the blood stream, but this in turn may limit the efficiency of release once at the tumor site. Other studies have shown that by creating an ADC using a small immunoprotein (SIP) antibody (small immunoprotein, comprised of an IgG, including variable regions from heavy and light chains linked through peptide plus additional C3 or C4 heavy chain proteins; see also below) and comparing the results with an analogous ADC constructed with intact IgG, the release of the drug by the ADC-SIP occurs faster. This is probably due to a more stable interchain disulfide bond in the SIP. However, by analyzing the stability of ADCs in mouse plasma, a half-life greater than 48 h with IgG and less than 3 h with SIP was determined. An analysis of the in vivo efficacy of the above compounds showed that the ADC-SIP experienced an accumulation and therefore a greater release than the IgG-ADC, despite there being a global accumulation of ADC-IgG after 24 h that was greater in the tumor than that observed for the ADC-SIP [23].

##### Cathepsin B-Sensitive Linker

**Figure 4:**
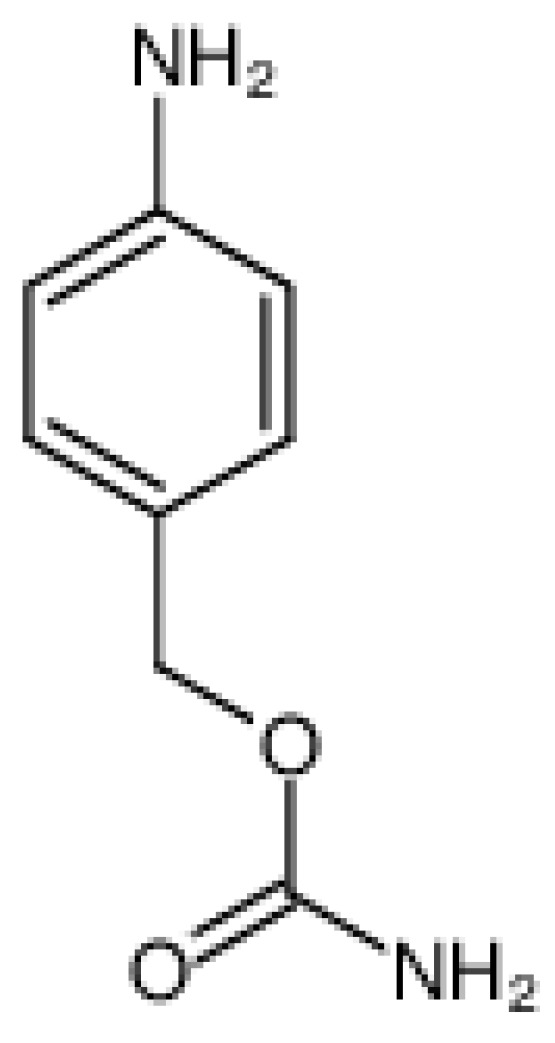
PABC, p-aminobenzyl carbamate, CAS#:918132-66-8.

The cysteine protease Cathepsin B is normally found inside late endosomes and lysosomal compartments in mammals. It is also implicated in tumor progression, being overexpressed by many cancers [36]. The carboxydipeptidase activity of Cathepsin B allows the splitting of a dipeptide linker that can bind a payload to the terminal C. This enzyme has various substrate target peptide sequences with Phe-Arg being the most common [36]. In addition, it also preferentially recognizes sequences such as valine-citrulline (Val-Cit) and phenylalanine-lysine (Phe-Lys) where the protease breaks a peptide bond on the C-terminal side of Val-Cit, Val-Ala, or Phe-Lys. Some studies have shown that a high pH basic environment increases the cleavage capacity [3] and that the hydrophobic residues Phe, Val, and Ala allow cleavage with cathepsin B that has the effect of increasing the stability in plasma. Sometimes, however, the payload can be too bulky in which case the use of a spacer that is stable and that does not alter the drugs chemistry, and functionality is necessary. One of the most used conjugation reagents is para-aminobenzyl carbamate (PABC) (Figure 4), that possesses a self-cleavage ability allowing it to release the unmodified payload [35]. For example, linkers containing Phe-Lys-PABC and Val-Cit-PABC, used for ADC with monomethyl-auristatin E (MMAE) payload, have a half-life in plasma for Phe-Lys-PABC of 12 h compared to 80 h for Val-Cit-PABC 80 h. This shorter half-life indicates that the linker with Phe-Lys-PABC is probably non-specific with the danger that it may exert off-target toxicity [37].

To summarize, it has been shown that if these types of linker are coupled with paminobenzyloxycarbonyl (PABC) they work more efficiently as cleavable linkers (i.e., Val-Ala-PABC) for ADCs [38]. The PABC group acts as a spacer separating the toxic payload from Val-Cit sequence so that the active site of cathepsin B can gain better access to the cleavage sequence, thus, more effectively exploiting its protease activity, particularly if a large molecular sized payload is used. PABC is furthermore a self-immolate linker that, upon Cathepsin B cleavage, can undergo hydrolysis releasing the free drug to which it is attached (i.e., monomethyl-auristatin E (MMAE)) [39,40].

##### Hydrazone Linker

Hydrazone linkers or other similar molecules that are pH-dependent, have quite a stable structure at neutral pH (i.e., in the bloodstream at pH 7.4) and are hydrolyzed when they reach an acidic cellular compartment such as the lysosome (pH < 5) or late endosomes (pH 5.5–6.2). However, the degradation of this linker is not confined to the lysosome, but may, on occasion, also occur extracellularly. ADCs with a hydrazone linker hydrolyze only slowly under physiological conditions, with the slow release of the toxic payload [41]. A study with an antibody directed against mucin, conjugated via an acid-labile linker, showed good therapeutic effects in a preclinical pancreatic cancer model [42] where the tumor microenvironment is significantly more acidic than in normal tissues, due to the enhanced glycolysis taking place in the tumor with the consequent production of lactate to a level sufficient to induce extracellular cleavage of the linker. In mouse models, the slow release of the circulatory payload has produced promising results, but only in the presence of payloads with moderate cytotoxic activity. Payloads with higher cytotoxic activity, now widely used for the production of ADCs, demand the use linkers with higher stability to avoid the undesired release of the payload and resultant non-specific systemic toxicity [37].

##### Glycosidase-Sensitive Linkers

Glycosidases comprise hydrolytic lysosomal enzymes, such as β-glucuronidases that degrade β-glucuronic acid residues into polysaccharides. They are found in lysosomes and work under hydrophilic environments. β-glucuronidases, like cathepsin B, are also secreted in the necrotic areas of some tumors. They are also enzymatically active in the extracellular environment [43]. ADCs that contain β-glucuronic acid can reach a DAR = 8 without causing aggregation and without reducing the hydrophobicity of the ADC. Indeed, this type of linker greatly reduce plasma clearance of ADCs, thus increasing their efficacy in vivo [44]. It is also established that the use of Poly (Ethylene Glycol) PEG linkers increases the hydrophilicity of β-glucuronic acid and, thereby, increases the activity and efficiency of the ADC [30].

Another type of hydrolytic lysosomal enzyme, the β-galactosidases that degrade β-galactoside, are also overexpressed in some types of cancer [45]. An ADC based on trastuzumab linked to MMAE using a β-galactoside linker was shown to be more potent than an equivalent ADC based on a Val-Cit-PABC linker. This formulation of ADC-β-galactoside-DM1 has also been shown to be more efficient in vivo for the treatment of HER2+ breast tumors than the approved trastuzumab emtansine (T-DM1) [35].

#### 2.2.2. Non-Cleavable Linkers

The most used non-cleavable linkers are alkylic and polymeric. For example, the MCC amine-to-sulfhydryl bifunctional cross-linker contains a cyclohexane ring structure that through steric hindrance protects the resulting thioether bond from hydrolysis [46]. The greatest advantage of non-cleavable versus cleavable linkers is their improved plasma stability; that results in reduced off-target toxicity in comparison to conjugates with cleavable linkers and thus provides greater stability and tolerability [47,48]. It is noteworthy that non-cleavable ADCs often have less activity against tumors due to the heterogeneity of target antigen expression where a bystander effect is an important contributor to therapeutic efficacy [49]. As described earlier, non-cleavable linkers require mAb degradation within the lysosome after ADC internalization to release the drug to the site of pharmacological activity in the cytosol. If the payload is linked to a charged amino acid such as lysine) with a Pi < 9.5, this will prevent escape of the drug by diffusion through the cell membrane and result in higher levels of drug-accumulation in the tumor cell which as a consequence should overcome the limitations of any bystander effect. In summary the major advantage of non-cleavable linkers is that they minimize drug release into the circulation thus limiting non-specific toxicity whilst maintaining, good in vivo stability [50].

Usually, non-cleavable linkers contain a thioether or maleimidocaproyl group. Examples of non-cleavable linker-based ADCs containing monomethyl auristatin F (MMAF), an anti-mitotic drug, where it was demonstrated that the drug is more potent if linked via a simple alkyl chain to the antibody. Conjugation effected with a non-reducible thioether linker demonstrated very good activity in both *in vitro* and in vivo [51].

### 2.3. Payloads

Currently, most ADCs are constructed with two main families of highly toxic compounds, acting either on microtubule or DNA structure. Among the first group, auristatins and maytansines payloads both act as tubulin inhibitors and have been widely used for construction of ADCs. Both molecules are potently cytotoxic against rapidly dividing cancer cells and have reduced toxicity to normal cells. Alternatively, calicheamicins and PBDs are DNA-damaging agents, inducing cell death by apoptotic mechanisms in all cells including cancer stem cells (CSCs), and for this reason, they do exert severe side effects. There is also a third category of drug that targets specific enzymes essential for cell survival. In general, the payloads suitable for an ADC must have: (a) good solubility in aqueous solutions allowing an easier conjugation to the antibody and ensuring enough solubility to ADC under physiological conditions; (b) a significantly higher cytotoxic activity (half maximal inhibitory concentration (IC_50_) ranging from 0.01 to 0.1 nM) in comparison to clinically standard chemotherapeutic agents; (c) induce cancer cell death by apoptotic mechanisms; and (d) possess an appropriate functional group to facilitate conjugation to the antibody.

**Figure 5:**
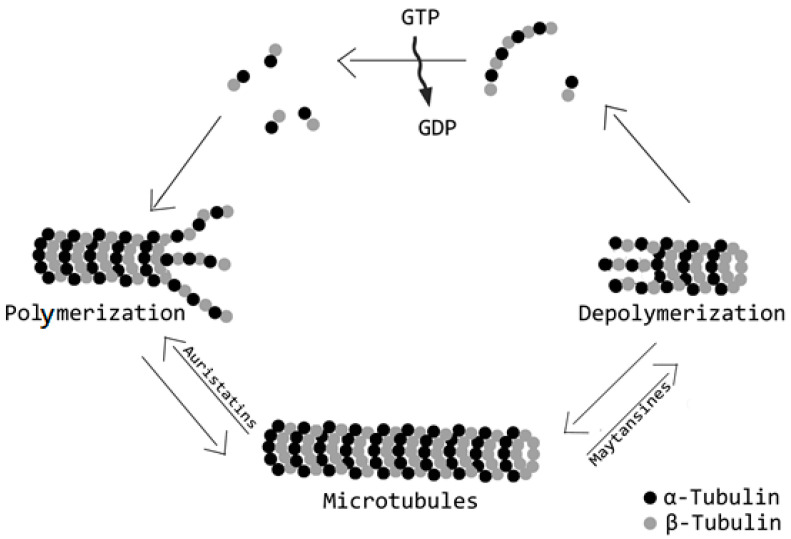
Mechanism of action of tubulin inhibitors payloads: polymerization promoters (microtubule stabilizers) and tubulin polymerization inhibitors (microtubule destabilizers). In the figure are two exemplifying drugs acting on microtubule formation: auristatins alters the formation of microtubules by binding on the β-subunit of α-β tubulin dimers; thus, producing uncontrolled growth of microtubules. Maytansines, on the contrary, stop tubulin dimers formation impairing the production of mature microtubules.

**Figure 6:**
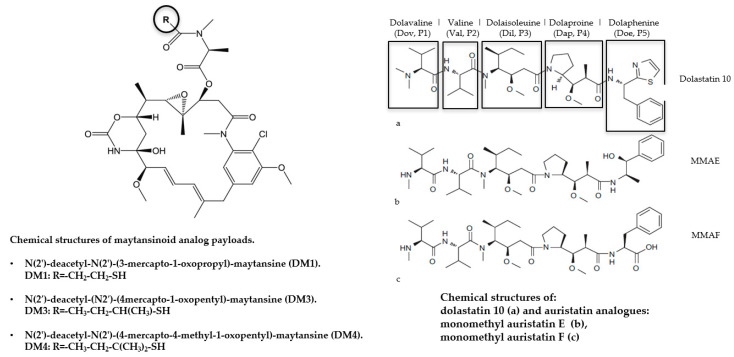
Classical microtubule-targeting agents: maytansinoids (**left**) and auristatin families (**right**).

The most widely used commercialized drugs for ADC formulation comprise microtubule-targeting agents. The choice of tubulin inhibitors as payloads is appropriate since rapid cellular proliferation is one of the major discriminating features between cancerous and normal cells and antimitotic agents are in principle less toxic to the normal cells [52]. Vinca alkaloid, laulimalide, taxane, maytansine, and colchicine have all defined binding sites on microtubules. These molecules (Figure 5) can be grouped in two main categories depending on their mechanism of action: tubulin polymerization promoters (microtubule stabilizers) and tubulin polymerization inhibitors (microtubule destabilizers) [53]. In particular, microtubule stabilizers inhibit the formation of microtubules acting on the β-subunit of α-β tubulin dimers determining unregulated microtubule growth, as in case of Auristatin. In contrast, the mechanism of action of microtubule destabilizers is to block the polymerization of tubulin dimers by inhibiting the formation of mature microtubules, as is the case for maytansinoids (Figure 6).

Auristatin is a dolastatin synthetic analog. The original drug was isolated from *Dolabella auricularia* (sea hare) as dolastatin peptides, which successfully improved its water solubility to give auristatin [54]. Auristatins block tubulin assembly and induce cell cycle arrest in G2/M phase, causing cells to undergo apoptosis.

To prevent lysosomal payload degradation and to enhance drug efficacy two innovative auristatin derivatives (monomethyl auristatin E (MMAE) and monomethyl auristatin F (MMAF)) have been developed by Seattle Genetics. These two compounds are synthetic drugs derived by design from structure-activity relationship (SAR) analysis. These two new molecules are different due to a phenylalanine present at the C-terminus of MMAF that allows this latter compound to be more membrane impermeable. In contrast, MMAE can exit the cell and thus diffuse to nearby cells killing them through bystander effects [53].

Maytansinoids are derivatives of natural cytotoxic agents named maytansines, a family of toxins originally isolated from the cortex of *Maytenus serrata* possessing macrolide structure. Maytansine and maytansinoids alter microtubule polymerization thus inhibit the maturation of microtubules by binding to or in close proximity to the vinblastine-binding site on the β-subunit of tubulin. This consequently induces cell death through mitotic arrest [54].

ADCs that containing maytansinoid, are unfortunately substrates for multidrug resistance protein 1 (MDR1), a critical protein of the cell membrane that acts by actively pumping a wide variety of xenobiotics out of cells. To prevent this problem a series of hydrophilic linkers have been used in ADC chemistry. These linkers allow for an increased drug content (DAR) in ADCs s and subsequent increases in the amount of drug delivered to each target cell. The increased polarity introduced by such linkers allows the formation of maytansinoid metabolites that are poor substrates for efflux pumps thus overcoming MDR [55].

Maytansines are difficult to conjugate because they do not have reactive chemical groups. To overcome this problem, a series of derivatives containing SH groups have been created examples of which are, DM1 and DM4 that are substituted by methyl disulfide at the maytansine C3 *N*-acyl-*N*-methyl-l-alanyl ester side chain [56].

**Figure 7:**
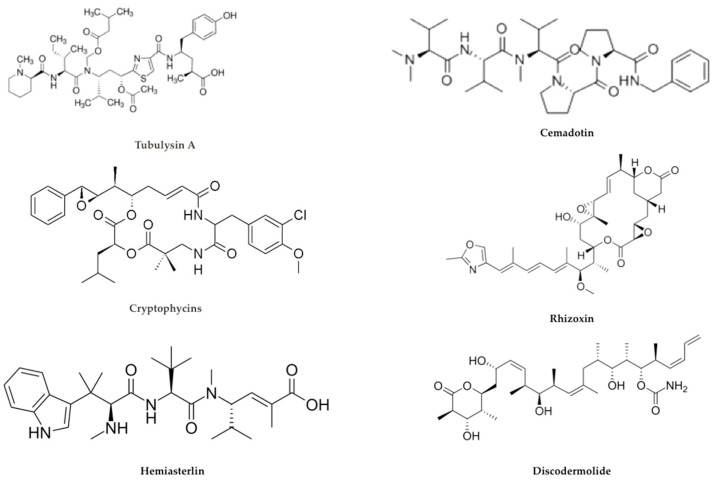
Other microtubule-targeting agents.

A third type of antimitotic payload includes tubulysins characterized by higher affinity of binding to the vinca domain of tubulin if compared with vinblastine. These agents exert a rapid disruption of the cytoskeleton and subsequent disassembly of the mitotic apparatus in proliferating cancer cells. This results in a block at G2/M of the cell cycle and subsequent apoptotic cell death [57] (Figure 7).

Tubulysins possess high degree of selective cytotoxicity against human cancer cells due to their rapid rate of division. Furthermore, they may also be effective against cancer cells overexpressing the P-glycoprotein or which possess mutations in tubulin gene. Tubulysins are comprised of a family with 14 different isoforms characterized by conserved core structure made of an L-isoleucine (Ile), a tubuvaline (Tuv), and an *N-*methylD-pipecolic acid (Mep) unit.

The first targeted drug (EC0305) based on tubulysin has been recently obtained by linking Tubulysin B to folic acid conjugate. Now several tubulysin D-based ADCs are under study [53].

To complete the family of drugs that bind to microtubules the following compounds are also worth mentioning:

Cryptophycins, a class of cytotoxins more potent than MMAE and DM1, isolated from *Nostoc cyanobacteria* induce tubulin depolymerization binding to microtubules. Cryptophycin-1 is the main component, acting on many solid tumors and additionally MDR cancer cells.

Hemiasterlin from marine sponges are naturally occurring tripeptides acting as potent inhibitors of cell growth. They bind to the tubulin vinca-site thus disrupting normal microtubule dynamics and consequently inhibiting tubulin polymerization. Taltobulin (HTI-286) is a fully synthetic analog of hemiasterlin and has been shown to be to be active against a variety of MDR cancer cell lines [53].

Cemadotin (LU103793) is a more hydrophilic synthetic pentapeptide analogous of dolastatin 15, possessing strong antiproliferative activity through inhibition of microtubule assembly and tubulin polymerization by binding at a novel site on tubulin. Cemadotin has been shown to be an effective payload for ADC construction [53].

Rhizoxin, a compound isolated from Rhizopus microspores (a fungus able to be infectious for humans causing mycosis) that binds to tubulin and causes inhibition of microtubule assembly [58].

Discodermolide is so far the most efficient natural promoter of tubulin assembly considered to be a very promising candidate for future ADC development [53].

There are furthermore other tubulin inhibitors that have been investigated for their possible use in ADC construction, such as taccalonolide A or B, taccalonolide AF or AJ, colchicine, epothilone A and B, taccalonolide AI-epoxide, CA-4, laulimalide, paclitaxel, and docetaxel, together with their synthetic analogous [53,59].

The second category of payload used for ADC construction is comprised of DNA-damaging drugs. This class of payload may be more effective than microtubule inhibitors with IC_50_ values in the picomolar, as opposed to the nanomolar range for microtubule inhibitors. This would make ADCs constructed with DNA damaging drug payloads more potent and therefore better suited for targeting antigens that are expressed at low levels on tumors. Furthermore, DNA-damaging drugs are fully capable of apoptotically killing non-dividing cells including cancer stem cells when used in combination with drugs that inhibit DNA repair and furthermore are capable of killing target cells at any point in the cell cycle [60].

**Figure 8:**
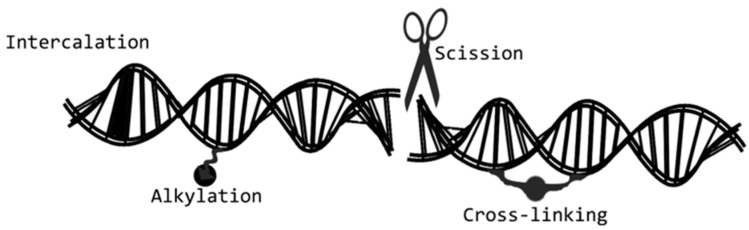
The four main mechanisms of action of DNA-damaging agents: DNA double-strand breakers, DNA alkylators, DNA intercalators, and DNA cross-linkers. DNA-damaging agents. These drugs can act at any phase of tumor cell life cycle.

There are at least four mechanisms of action exerted by DNA-damaging agents, which are as follows: (a) DNA double-strand breakage, (b) DNA alkylation, (c) DNA intercalation, and (d) DNA cross-linking. The most used DNA-damaging payloads are pyrrolobenzodiazepine, duocarmycins, doxorubicin, and calicheamicins [61] (Figure 8).

Pyrrolobenzodiazepines (PBDs) were originally isolated from *Streptomyces* sp. and are natural products, possessing antibiotic and antitumor properties. PBD molecules bind in the minor groove of double- stranded DNAs to the C2-amino groups of guanine residues.

PBDs forms an adduct PBD/DNA in the minor groove of DNA, leading to decreased DNA repair and interfering with transcription factors binding to DNA, as well as to some enzyme functions including RNA polymerase and endonucleases.

**Figure 9:**
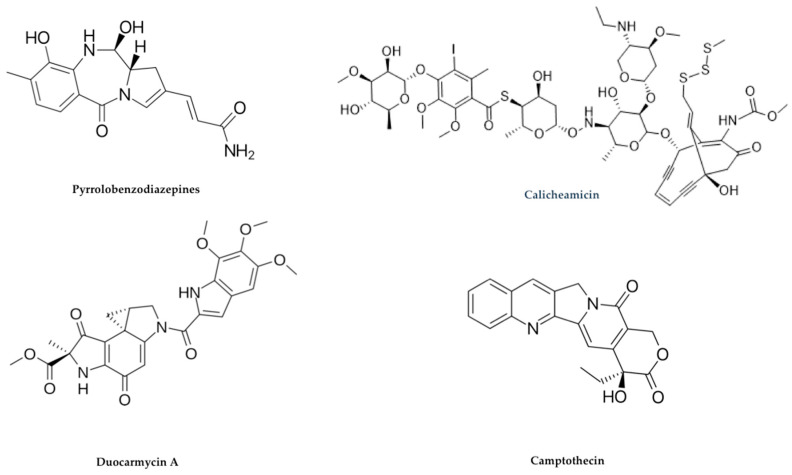
Examples of DNA-damaging drug payloads.

Currently, additional to natural isolated monomeric forms of PBDs, synthetic PBD dimers are available, which in addition to forming monoadducts are also capable of forming intrastrand or interstrand DNA cross-links [62] (Figure 9).

Duocarmycins, metabolites originally isolated from *Streptomyces* sp. are powerful cytotoxic substances because their mechanism of action involves alkylation of the DNA minor groove to form a stable adduct. Duocarmycins specifically bind to a sequence of five-base-pair rich in AT-rich where the central pyrroloindole may be easily accommodated. This results in irreversible DNA modification compromising its architecture that finally leads to DNA cleavage and apoptotic cell death. There are also synthetic analogs of duocarmycins available, such as adozelesin, bizelesin, and carzelesin.

Duocarmycins have impressively high cell cycle-independent cytotoxicity against a variety of proliferating cancer cells *in vitro* with IC_50_ values in the pM range [63].

The duocarmycin analogous DUBA (duocarmycin-hydroxybenzamide-azaindole), representing the duocarmycin final active drug metabolite, has been used to produce different new-generation ADCs that have been tested *in vitro* and in vivo to verify their therapeutic efficacy. An example is represented by SYD983, an anti-HER2 ADC, exerting clear anti-tumor activity in a mouse xenograft model (BT-474) and showing enough stability in human and macaque primate plasma [64].

The high toxicity of duocarmycins and their analogous makes them desirable candidates to maximize ADC cell-killing activity and also suggests that they may be effective agents to overcome multi drug resistant (MDR) tumor cells [65].

Calicheamicins (LL-E33288) are a class of antibiotics that were discovered in Texas following a search for novel fermentation-derived antitumor antibiotics that led to *Micromonospora echinospora*. These compounds are a class of enediyne-containing DNA-cleaving antitumor agent with a potency 4000–10,000 times greater than DNA intercalating drugs, such as Adriamycin and other similar.

The mechanism of action of calicheamicins after cell entry and nuclear diffusion is due to drug targeting and binding to the minor groove of DNA, causing double-strand breaks that induce apoptotic cell death [66].

Calicheamicins are extremely powerful drugs acting at sub-pM concentrations but also unfortunately exert significant non-specific toxicity, damaging the DNA of all cells. Their high toxicity means that they cannot be used directly as a single therapeutic agent in cancer treatment. Their inherent characteristics (i.e., high cytotoxicity, relatively small molecular size, mechanism of action) have however made calicheamicins useful payloads for the construction of ADCs [54].

Camptothecin (CPT) is a natural compound isolated from *Camptotheca acuminata* and is an inhibitor of the nuclear enzyme topoisomerase I. CPT molecules inhibit both DNA and RNA synthesis in mammalian cells, and have demonstrated to be strongly cytotoxic against a wide range of experimental tumors. Unfortunately, several clinical trials have shown considerable toxicity problems in patients due to their low solubility and resultant adverse side effects. To circumvent these limitations, camptothecin analogs topotecan (TPT) and irinotecan (camptothecin-11, CPT-11) that show improved water solubility have been approved by the FDA. These molecules were tested in clinical practice, and demonstrated significant antitumor activity and reduced toxicity [67].

SN-38 and DX-8951f are two additional CPT-analogs that have been used as ADC payloads. SN-38, an active CPT-11 metabolite that exploits inhibition of DNA topoisomerase to exert its anticancer activity [68].

In addition to all of the above-mentioned payloads, other molecules available also act as DNA-damaging agents for incorporation into newly emerging ADCs. Among these compounds, particular mention should be given to iSGD-1882 (DNA minor groove cross-linker derived from PBD dimers), centanamycin (binds to DNA and alkylates or intercalates into DNA), PNU-159682 (an anthracycline metabolite) [69], and uncialamycin (an enediyne natural product isolated from Streptomyces uncialis) [70], all active on different cancer cell lines, and finally indolinobenzodiazepine dimers (IGNs) bind to the DNA minor groove leading to DNA cross-linking [71].

#### Alternative Payloads

In addition to all the payloads discussed above, other molecules are available whose cytotoxicity is based on different mechanisms of action that include the direct induction of apoptosis, spliceosome, and RNA polymerase inhibition.

Bcl-2 family members, including Bcl-xL, are overexpressed in cancer and the BH3- binding domain on Bcl-xL has been targeted. Examples of such targeting agents comprise two anti-EGFR-Bcl-xL ADCs both of which possessed reasonable anti-tumor activity [72].

The spliceosome is an attractive target in cancer therapy, and thailanstatins have been shown to inhibit RNA splicing by the binding to different spliceosome subunits [61]. Thailanstatin A in fact was demonstrated to bind to the SF3b subunit of the spliceosome blocking RNA splicing and was used in the generation of an ADC (anti-Her2-thailanstatin). The Spliceostatins are potent spliceosome inhibitors of natural origin with interesting and potentially useful anticancer activities [61].

The final class of promising payloads are the transcription inhibitors targeting RNA polymerase II. Example of these compounds are the amatoxins, macrocyclic peptides produced by mushrooms of the genus Amanita, that are powerful and selective inhibitors of RNA polymerase II, thus resulting in the inhibition of protein synthesis [73].

β-amanitin has been covalently coupled to a MUC1-targeting mAb and this ADC has proven to be specifically cytotoxic against the human breast carcinoma cell lineT47D [74].

α-amanitin was efficiently targeted to cancer cells through an anti-HER2 mAb, with an IC_50_ value in the pM range. Moreover, α-amanitin has also been covalently linked to an EpCAM-targeting mAb, showing effective antiproliferative activity both *in vitro* and in vivo. An anti-PSMA-α-amanitin ADC has been recently observed to have in vivo antitumor activity when coupled using a stable and cleavable linker [56].

Amatoxins are highly water soluble, a property that facilitates the conjugation process and reduces ADC aggregation. Their low molecular weight, after release, allows for rapid kidney excretion in the urine. Amatoxins are also highly active against MDR cancer cells because they represent poor substrates for MDR mechanistic processes [71].

It should also be mentioned that payloads for conjugation to antibody can also include proteinaceous enzymes from plants (e.g., saporin, ricin A chain) [4,20] or bacterial toxins (PE, Pseudomonas exotoxin, DT, Diphtheria toxin) which induce cell death by irreversibly inhibiting protein synthesis catalytically [75,76]. Although this latter class of toxin molecule when conjugated to an antibody is commonly known as an immunotoxin, it is not considered a small molecule drug. The enzymatic nature of proteinaceous toxins as a payload represents added value since a single molecule may be sufficient to fatally intoxicate an individual cell. A variety of different linkers and payloads has been investigated over the years and because these are totally protein constructs, fully recombinant toxins are possible making this a promising production strategy [4].

**Figure 10:**
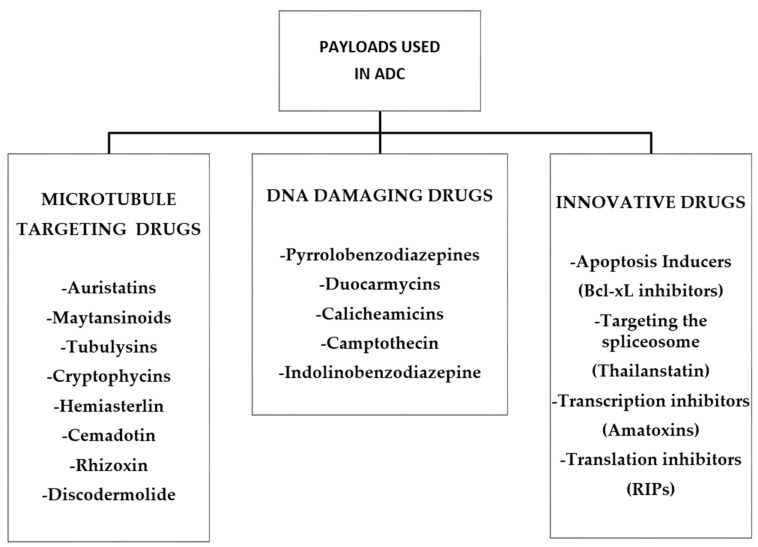
Summary diagram of the different classes of cytotoxic molecules used in ADC construction.

The Figure 10 below summarizes all the payload categories discussed above in Section 2.3.

## 3. Conjugation Strategies

**Figure 11:**
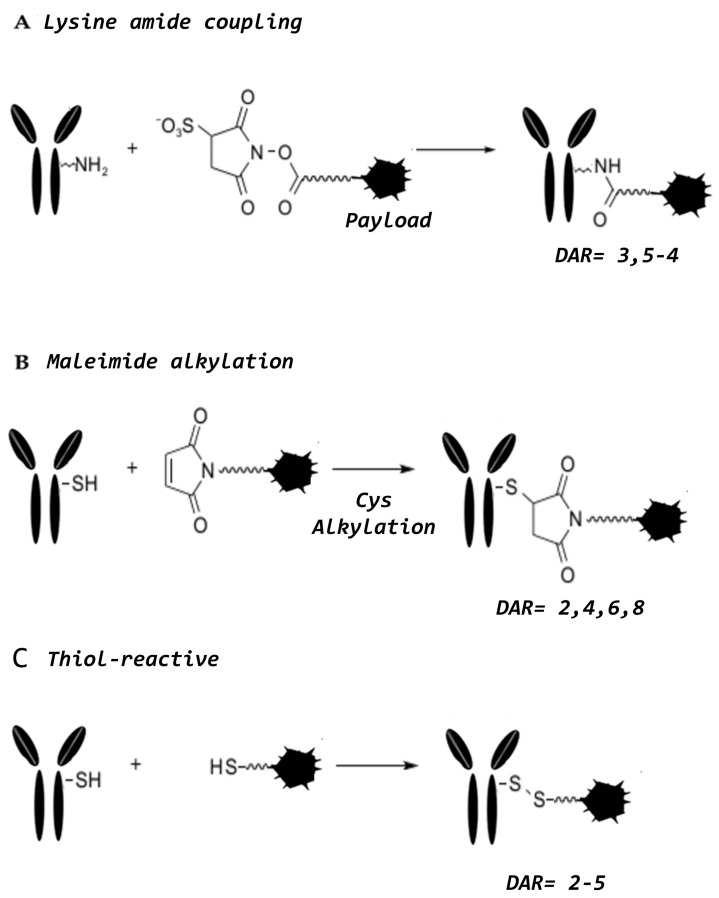
Main reactions used in the cross-linking procedures. (**A**) Lysine amide coupling, (**B**) Maleimide Alkylation, and (**C**) thiol-reactive conjugation.

Most ADCs exploit the presence of lysine and cysteine residues within the polypeptide structure of the antibody as the point of conjugation. The average IgG_1_ molecule for example, possesses approximately 90 lysine residues, but only 30 of these are accessible for conjugation, so theoretically the number of covalently coupled payloads could range from 1 to 30. Amide or amidine bond formation on the side chain of lysine is the most common reaction to effect covalent cross-linking of the antibody to the payload through exploitation of the reactive groups of linkers (i.e., *N-*hydroxysuccinimide esters, NHS; imidoesters) [77]. Figure 11 shows the main reactions used in the cross-linking procedures.

The lysine-amide coupling conjugation is not site-specific and requires a pre-conjugation derivatization of the antibody and/or the payload in order for conjugation to proceed, very often using thiolic or citrulline-valine as linkers [77]. Alternatively, conjugation via cysteines requires that a partial reduction of the antibody is undertaken or a thiol-containing reagent (e.g., Trout’s reagent) is used to introduce additional-SH groups available for the conjugation. This may cause destabilization of the whole IgG molecule and introduce structural heterogeneity into the final product. IgG_1_ has four disulfide bridges, two that link the heavy to the light chains, and two in the hinge region, which bond together the two half-heavy chains of the whole antibody [78]. As one of the fundamental points of conjugation chemistry is the precise control of the drug Antibody Ratio (DAR), a recently used strategy is to achieve site-specific coupling of the payload by engineering the structure of the antibody. For example, the substitution of three cysteines in the hinge region with three serines yields an IgG molecule that fully retains its interactions between heavy and light chains [79]. Thus, through such modification of the cysteine residues, this leaves only two reactive cysteines, one on each chain, to yield an ADC product containing up to two molecules of drug per antibody. By refining the conjugation conditions, it is possible to obtain an extremely homogeneous product with the presence of the only conjugate with exactly two molecules of drug per antibody molecule (DAR 2) [23,34,79].

## 4. Site-Specific Enzymatic Conjugation

It is possible to use enzymatic methods to perform a site-specific controlled conjugation. This requires enzymes that react with the antibody and can induce a site- or amino acid sequence-specific modification. The most used enzymes are: sortase, transglutaminase, galactosyltransferase, and syaliltransferase. Sortase A from *Staphylococcus aureus* binds the LPXTG sequence and breaks the bond between glycine and threonine linking an oligoglycine (oligo-G) that can be used to bind the desired payload. A transglutaminase derived from *Streptomyces mobaraensis* catalyzes transpeptidation and recognizes an LLQG motif that has been inserted into a genetically engineered antibody, giving rise to a convenient site-specific ADC conjugation site. An application of a transglutaminase conjugation method gave rise to improvements in DAR for ADCs constructed with a branched linker that allowed for the loading of multiple payload molecules. Anami and coworkers developed an innovative conjugation method utilizing a branched linker on an anti-HER2 monoclonal antibody with MTGase, without a reduction in antibody binding affinity leading to the production of a homogeneous ADC molecular population with a remarkable increased DAR (up to 8) using monomethyl auristatin F as the payload [80].

The linkers used contain a lysine scaffold to generate a branch point and PEG spacers to increase ADC mobility. For MTGase-mediated antibody-linker conjugation, the presence of a primary amine is necessary as well as the presence of two reactive azide groups to link with the payloads [46]. Using MTGase this azide-linker can be bound to the glutamine residue Q295 in the IgG heavy chain. To generate an ADCs with DAR 2 the chosen payloads can be bound through azide-alkyne cyclization using a linear or branched linker to generate a DAR 4 ADC. This improved ADC showed increased *in vitro* cytotoxicity against HER2-expressing breast cancer cell lines compared to ADCs produced by more traditional methods [46].

An *N-*Glycan engineering strategy takes advantage of conserved Asn297 (N297) within the Fc domain in all IgG classes. In order to create a reactive aldehyde group on the *N-*glycan terminus it is possible to employ either β-1,4-galactosyltransferase (GalT) or α-2,6-sialyltransferase (SialT) enzymes to achieve this. The aldehyde groups enzymatically created are then used to conjugate amino-oxy-functionalized payloads [80]. Recently Bruins and coworkers used a mushroom tyrosinase to oxidize the exposed tyrosine residues on antibody to generate a 1,2-quinone, which can then be subjected to a nucleophilic reaction with thiols or amines from the side chains of amino acids such as cysteine, lysine, histidine, or any thus modified payload [81]. A further new recent strategy to improve ADC stability is site-specific conjugations using antibody engineered to incorporate non-natural amino acids (nnAA). The introduction of nnAA with orthogonal reactive functional groups (aldehyde, ketone, azido, or alkynyl tag) increases the homogeneity of ADCs and enables novel bioorthogonal chemistry that utilizes reactive groups that are different from the classical thiol or amine reactions. The most used nnAA or strategies are: seleno-cysteine, p-azidomethyl-L-phenylalanine (pAMF) p-acetyl phenylalanine (p-AcPhe), FGE (formylglycine generating enzyme) azide or alkynyl nnAA or glycan. To improve ADC stability, Transtuzumab was engineered to introduce p-AcPhe that could react through the carbonyl group (ketone) with a drug containing an alkoxy-amine to produce an oxime [82]. To achieve this, engineered new cell lines or cell free protein expression systems (OCFS: Open Cell Free Synthesis) were generated that possess the transcriptional machinery capable of inserting the a nnAA exactly where desired. In this system, the most important element needed for nnAA incorporation is a aminoacyl tRNA synthetase (aaRS) that charges a specific tRNA with the nnAA [83,84].

## 5. Approved ADCs and Future Perspectives 

The ADC gemtuzumab ozogamicin, also known with the commercial name of Mylotarg^®^ produced by Pfizer Inc., was the first ADC approved twenty years ago by the U.S. Food and Drug Administration (FDA). Mylotarg^®^ was used to target the CD33 (Cluster of differentiation 33, sialic acid binding Ig-like lectin 3 (Siglec3)).

myeloid associated leukocyte differentiation antigen expressed by myeloid leukemia cells (CD33^+^ AML). Currently, Mylotarg^®^ is indicated for the treatment of patients diagnosed since at least two years with recurrent or refractory CD33^+^ AML [85].

The Mylotarg^®^ ADC was produced using a random conjugation technique with an amide bond interposed between the cleavable linker, hydrazone acetyl butyrate with the antibody attached to the calicheamicin payload via a lysine sidechain on the antibody [86]. The history of its approval has been complicated due to unexpected toxicities, in particular veno occlusive disease (VOD) in the liver in a significant proportion of patients. Myelotarg was initially approved by the FDA in the USA in 2000 but then voluntarily withdrawn from the market in 2011 following toxicity-related deaths and a lack of good clinical evidence showing its therapeutic benefits. Subsequently however, lower dose studies have demonstrated its safety and have clearly shown it to be of clinical benefit in a subset of AML patients [87].

In 2017, Myelotarg was once again approved by the FDA [88] and immediately following this approval another calicheamicin-based ADC using the same linker technology (linker-antibody bond and cytotoxin, bystander effect) inotuzumab ozogamicin (also known as Besponsa^®^) directed against the B-cell restricted differentiation antigen CD22 [89]. Besponsa^®^, was approved for use in the EU for the treatment of acute lymphoblastic leukemia currently under orphan drug status [90,91].

The second US, EU, and Japan approved ADC was brentuximab vedotin (Seattle Genetics, Inc. and Takeda Pharmaceutical Company Ltd.). The commercial name for this ADC is ADCETRIS^®^ (Seattle Genetics Inc., n.d.) and is indicated for the treatment of Hodgkin’s lymphoma targeting the Reed-Sternberg cell-associated antigen, CD30. This ADC was constructed using a protease-cleavable mc-VC-PABC linker and Monomethyl auristatin E (MMAE) as the cytotoxic drug payload [92]. The chemistry of linking method to provide a bystander effect is achieved through a dithiol bond via to a cysteine residue on the antibody. Adcetris^®^ (brentuximab vedotin) has been approved by FDA in 2011 [93].

The final and most recent approved ADC at the time of writing is trastuzumab-emtansine (Roche Registration GmbH) sold under the commercial name Kadcyla^®^. The Trastuzumab (commercial name Herceptin) is a monoclonal antibody used as a naked antibody to treat HER2^+^ breast cancer by targeting the antigen HER-2 (Human Epidermal growth factor Receptor) and triggering host-mediated antibody dependent cellular cytotoxicity (ADCC) while simultaneously downregulating EGFR-mediated growth signaling thereby inhibiting tumor growth [94]. The ADC Kadcyla^®^ uses a maytansinoid derivative as the anti-neoplastic drug payload (DM-1) and a non-cleavable SMCC (amide antibody-linker) as linker. This ADC shows reduced bystander effect, strongest activity compared to Herceptin in certain conditions [86] and has been approved in the US, EU and Japan since 2013 [95,96,97].

Over the past two years, the FDA approved two new ADCs: Polivy^®^ (Polatuzumab vedotin) and Lumoxiti^®^ (Moxetumomab pasudotox). The Polivy^®^ is a humanized monoclonal antibody, directed against CD79B (an antigen expressed by Large B-Cell lymphoma). Polivy is the first commercial therapeutic ADC produced using a site-specific covalent bond conjugated to the synthetic monomethyl auristatin E (MMAE) via engineered cysteines (THIOMABs) and using a protease-cleavable peptide linker to increase the plasma stability [98].

The Lumoxiti^®^ is the first approved recombinant ADC. It is an innovative linkerless ADC is produced by genetic fusion between the Fv fragment of an anti-CD22 monoclonal with the 38 kDa fragment (PE38) of Pseudomonas exotoxin A [99].

We can underline that all the above-described approved ADCs (except the unique recombinant linkerless ADC Lumoxiti^®^) were developed using conventional random conjugation methods [100]. Table 1 reports shows all the approved and marketed ADCs.

## 6. Future Perspectives

The approved ADCs are mostly indicated for the treatment of hematological malignancies and, with a few exceptions, their clinical activity has largely failed for solid tumors. The reasons for these failures may be attributed to the large molecular size of the ADC molecule that shows poor penetration into the tumor mass, thus resulting in poor in vivo efficacy [11]. For this reason, other forms of reduced sized antibodies such as single chain fragments of variable regions (scFv), i.e., v regions joined by a linker peptide, have been investigated, Also in the form of heterodimers of IgG and IgE, a small divalent immunoprotein (SIP, 75 kDa) or “minibody”, a homodimer stabilized by a disulfide bond to its C-terminal [13]. The most explored antibody derivative variants are the dsFv and scFv. They are made of V_h_ and V_L_ domains linked through an interchain disulfide bond (dsFv) genetically engineered and linked covalently with a hydrophilic linker to form an scFv. Due to their modular nature, they can undergo multimerization into homo and hetero oligomers (diabody, triabody, tetrabody) strengthening antigen binding affinity and diversifying the different functionalities. The sdAbs (single domain antibodies) are smaller than scFvs, comprising 15-KDa V_h_, V_l_, or V_hh_ domains, also termed nanobodies, and containing the antigen domain in the terminal region of the hinge. Similarly, to scFv, these nanobodies can form homodimers increasing the binding affinity for the target antigen or formed into heterodimers with bispecific properties. Bispecific antibodies can interact simultaneously with two antigens on the same target cell, a property that potentially allows for an increase in the therapeutic window while decreasing the non-specific effects on non-target cells [101]. SIP antibodies have high affinity to their antigen and their turnover occurs in the liver. The technology for producing SIP antibodies was developed by Neri et al. [79] against fibronectin and other vascular antigens. These antigens, common in tumors, are stable and accessible. In addition, SIP have two C-terminal cysteines that allows a disulfide bridge with various payloads [102]. All these small fragments of antibodies as Fab, diabody and scFv, penetrate more rapidly into solid tumors but have a reduced serum half-life and undergo rapid renal elimination. This means that they are often eliminated before adequate absorption takes place at the tumor site.

Depending on the tumor under treatment, it is necessary to adequately choose and modify the Fc portion on the antibody to have the best possible response, especially to take advantage of the effect of the ADCC combined with other mechanisms of cell killing exerted via direct antibody-mediated cell signaling [2].

In addition to the above-mentioned ADCs, there are also other constructs and strategies to attack cancer cells that involve the conjugation of toxins or chemotherapeutic drugs to ligands or proteins that are overexpressed on the target cell. The most used ligands as carriers can be proteins or peptides. Another strategy is to use peptide-drug-conjugates that are made up of small, synthetic peptides [103,104,105,106,107,108]. These molecules appear to have an even faster penetration and elimination than the small antibody fragments we have described [102].

Nanomedicine is one of the formulation-based technologies to increase bioavailability of drugs. Nanotechnology can provide new treatment options for tumors due to the great potential for selective targeting and controlled drug release. Increasingly more attention is being paid to antibodies and their fragments as targeting ligands able to bind specific receptors that are overexpressed on tumor cells [109] for the delivery of nanoparticles.

Non-targeted nanoparticles such as liposomal-based preparations [110] polymeric [111] and metallic nanoparticles [112,113] are readily available for the conjugation with antibodies and drugs, potentially opening the possibility to develop theragnostic (therapeutics and diagnostics) agents. These formulations can reduce the toxicity profiles of the payloads and improve the therapeutic widow. One example is Doxil1, which has been on the market for 20 years as a liposomal preparation of doxorubicin, and is now being improved by PEGylation [114].

Antibody conjugate nanoparticles (ACNPs) are formed from a combination of ADC and nanotechnologies. ACNPs similarly to ADCs use antibodies to specifically target cancer cells for the delivery of encapsulated drugs.

Many ACNPs have been tested in clinical trials, but to date none has yet reached phase III trials [115].

In recent years, great progress has been made in developing effective nanoparticle-based drug targeting using conjugated antibodies. In addition, the use of antibody fragments combined with advances in molecular design are overcoming some of the problems associated with the large molecular size of unmodified antibodies [109].

With the adoption of strategies that improve the ability of ACNP to reach the tumor site to facilitate active targeting together with additional studies that are still needed to define and refine conjugation technology, size, shape and surface charge of nanoparticles will likely lead in the future to useful outcomes for these targeting reagents.

## 7. Conclusions

More than 80 ADCs are currently under investigation and are in various stages of clinical development for cancer treatment [116]. Current evidence indicates that the field of ADCs is a very promising one, even though in past years they have faced a number of clinical failures. Recent advances in technology now provide all of the necessary elements required for the facile production of humanized monoclonal antibodies, site-specific conjugation protocols, various potent cytotoxic payloads with different mechanisms of action, adaptable linker technologies, together with advanced analytic techniques [117]. With the availability of the new technologies and biomarker selection strategies, ADCs are set to represent an important contribution to the future of immuno-oncology.

## Figures and Tables

**Table 1:** ADCs currently approved for clinical use.

Name	Antigen Target	Type of Cancer Target	Linker Type	Status
*Mylotarg^®^ (Gemtuzumab ozogamicin)*	CD33	Myeloid leukemia B-cell lymphoma	Cleavable linker (hydrazone acetyl butyrate)	marketed
*Besponsa^®^ (Inotuzumab ozogamicin)*	CD22	Lymphoblastic B leukemia	Cleavable linker (hydrazone acetyl butyrate)	marketed
*Adcetris^®^ (Brentuximab vedotin)*	CD-30	Hodgkin’s lymphoma	Protease-cleavable mc-VC PABC	marketed
*Kadcyla^®^ (Trastuzumab emtansine)*	HER-2	HER2^+^ Breast cancer	Non cleavable thioether linker	marketed
*Polivy^®^ (Polatuzumab vedotin)*	CD79B	Large B Cell lymphoma	Protease-cleavable	marketed
*Lumoxiti^®^ (Moxetumomab pasudotox)*	CD22	Refractory hairy cell leukemia	Recombinant covalently fused (linkerless)	marketed

## References

[1] Strebhardt K., Ullrich A. (2008). Paul Ehrlich’s magic bullet concept: 100 years of progress. Nat. Rev. Cancer.

[2] Hoffmann R.M., Coumbe B.G.T., Josephs D.H., Mele S., Ilieva K.M., Cheung A., Tutt A.N., Spicer J.F., Thurston D.E., Crescioli S. (2018). Antibody structure and engineering considerations for the design and function of Antibody Drug Conjugates (ADCs). OncoImmunology.

[3] Sochaj A.M., Świderska K.W., Otlewski J. (2015). Current methods for the synthesis of homogeneous antibody-drug conjugates. Biotechnol. Adv..

[4] Giansanti F., Flavell D.J., Angelucci F., Fabbrini M.S., Ippoliti R. (2018). Strategies to Improve the Clinical Utility of Saporin-Based Targeted Toxins. Toxins (Basel).

[5] Panowski S., Bhakta S., Raab H., Polakis P., Junutula J.R. (2014). Site-specific antibody drug conjugates for cancer theraphy. mAbs.

[6] Singh S.K., Luisi D.L., Pak R.H. (2015). Antibody-Drug Conjugates: Design, Formulation and Physicochemical Stability. Pharm. Res..

[7] Zhou Q. (2017). Site-specific conjugation for ADC and beyond. Biomedicines.

[8] Khongorzul P., Ling C.J., Khan F.U., Ihsan A.U., Zhang J. (2020). Antibody-Drug Conjugates: A Comprehensive Review. Mol. Cancer Res..

[9] Chari R.V.J., Miller M.L., Widdison W.C. (2014). Antibody-drug conjugates: An emerging concept in cancer therapy. Angew. Chem. Int. Ed. Engl..

[10] Weidle U.H., Maisel D., Klostermann S., Schiller C., Weiss E.H. (2011). Intracellular proteins displayed on the surface of tumor cells as targets for therapeutic intervention with antibody-related agents. Cancer Genom. Proteom..

[11] Gauzy-Lazo L., Sassoon I., Brun M.P. (2020). Advances in Antibody-Drug Conjugate Design: Current Clinical Landscape and Future Innovations. Slas Discov..

[12] Brüggemann M., Osborn M.J., Ma B., Hayre J., Avis S., Lundstrom B., Buelow R. (2015). Human antibody production in transgenic animals. Arch. Immunol. Exp. (Warsz).

[13] Aguiar S., Dias J., Manuel A.M., Russo R., Gois P.M.P., da Silva F.A., Goncalves J. (2018). Chimeric Small Antibody Fragments as Strategy to Deliver Therapeutic Payloads. Adv. Protein Chem. Struct. Biol..

[14] Sala G., Rapposelli I.G., Ghasemi R., Piccolo E., Traini S., Capone E., Rossi C., Pelliccia A., Di Risio A., D’Egidio M. (2013). EV20, a NovelAnti-ErbB-3 Humanized Antibody, Promotes ErbB-3 Down-Regulation and Inhibits Tumor Growth In Vivo. Transl. Oncol..

[15] Prasetyanti P.R., Capone E., Barcaroli D., D’Agostino D., Volpe S., Benfante A., van Hooff S., Iacobelli V., Rossi C., Iacobelli S. (2015). ErbB-3 activation by NRG-1β sustains growth and promotes vemurafenib resistance in BRAF-V600E colon cancer stem cells (CSCs). Oncotarget.

[16] Ghasemi R., Rapposelli I.G., Capone E., Rossi C., Lattanzio R., Piantelli M., Sala G., Iacobelli S. (2014). Dual targeting of ErbB-2/ErbB-3 results in enhanced antitumor activity in preclinical models of pancreatic cancer. Oncogenesis.

[17] Conner S.D., Schmid S.L. (2003). Regulated portals of entry into the cell. Nature.

[18] Kalim M., Chen J., Wang S., Lin C., Ullah S., Liang K., Ding Q., Chen S., Zhan J. (2017). Intracellular trafficking of new anticancer therapeutics: Antibody-drug conjugates. Drug Des. Devel..

[19] Rusten T.E., Vaccari T., Stenmark H. (2011). Shaping development with ESCRTs. Nat. Cell Biol..

[20] Capone E., Giansanti F., Ponziani S., Lamolinara A., Iezzi M., Cimini A., Angelucci F., Sorda R., Laurenzi V., Natali P.G. (2017). EV20-Sap, a novel anti-HER-3 antibody-drug conjugate, displays promising antitumor activity in melanoma. Oncotarget.

[21] Capone E., Lamolinara A., D’Agostino D., Rossi C., De Laurenzi V., Iezzi M., Sala G., Iacobelli S. (2018). EV20-mediated delivery of cytotoxic auristatin MMAF exhibits potent therapeutic efficacy in cutaneous melanoma. J. Control Release.

[22] Staudacher A.H., Brown M.P. (2017). Antibody drug conjugates and bystander killing: Isantigen-dependent internalisation required?. Br. J. Cancer.

[23] Dal Corso A., Gebleux R., Murer P., Soltermann A., Neri V. (2017). A non-internalizing antibody-drug conjugate based on an anthracycline payload displays potent therapeutic activity in Vivo. J. Control. Release.

[24] Mohamed M.M., Sloane B.F. (2006). Cysteine cathepsins: Multifunctional enzymes in cancer. Nat. Rev. Cancer.

[25] Lewis Phillips G.D., Li G., Dugger D.L., Crocker L.M., Parsons K.L., Mai E., Blättler W.A., Lambert J.M., Chari R.V., Lutz R.J. (2008). Targeting HER2-positive breastcancer with trastuzumab-DM1, an antibody-cytotoxic drug conjugate. Cancer Res..

[26] Dorywalska M., Strop P., Melton-Witt J.A.A., Hasa-Moreno A., Farias S.E., Galindo Casas M., Delaria K., Lui V., Poulsen K., Loo C. (2015). Effect of attachment site on stability of cleavable antibody drug conjugates. Bioconjug. Chem..

[27] Lu J., Jiang F., Lu A., Zhang G. (2016). Linkers Having a Crucial Role in Antibody-Drug Conjugates. Int. J. Mol. Sci..

[28] Chari R.V. (2008). Targeted cancer therapy: Conferring specificity to cytotoxic drugs. Acc. Chem. Res..

[29] Dorywalska M., Strop P., Melton-Witt J.A., Hasa-Moreno A., Farias S.E., Galindo Casas M., Delaria K., Lui V., Poulsen K., Sutton J. (2015). Site-Dependent Degradation of a Non-Cleavable Auristatin-Based Linker-Payload in Rodent Plasma and Its Effect on ADC Efficacy. PLoS ONE.

[30] Pillow T.H., Sadowsky J.D., Zhang D., Yu S.F., Del Rosario G., Xu K., He J., Bhakta S., Ohri R., Kozak K.R. (2017). Decoupling stability and release in disulfide bonds with antibody-small molecule conjugates. Chem. Sci..

[31] Wu B., Zhang G., Shuang S., Choi M.M. (2004). Biosensors for determination of glucose with glucose oxidase immobilized on an eggshell membrane. Talanta.

[32] Chari R.V., Martell B.A., Gross J.L., Cook S.B., Shah S.A., Blättler W.A., McKenzie S.J., Goldmacher V.S. (1992). Immunoconjugates containing novel maytansinoids: Promisinganticancer drugs. Cancer Res..

[33] Saito G., Swanson J.A., Lee K.D. (2003). Drug delivery strategy utilizing conjugation viareversible disulfide linkages: Role and site of cellular reducing activities. Adv. Drug Deliv. Rev..

[34] Giansanti F., Capone E., Ponziani S., Piccolo E., Gentile R., Lamolinara A., Di Campli A., Sallese M., Iacobelli V., Cimini A. (2018). Secreted Gal-3BP is a novel promising target for non-internalizing Antibody-Drug Conjugates. J. Control. Release.

[35] Bargh J., Isidro-Llobet A., Parker J., Spring D. (2019). Cleavable linkers in antibody–drug conjugates. Chem. Soc. Rev..

[36] Dubowchik G.M., Mosure K., Knipe J.O., Firestone R.A. (1998). Cathepsin B-sensitive dipeptide prodrugs. 2. Models of anticancer drugs paclitaxel (Taxol), mitomycin C and doxorubicin. Bioorganic. Med. Chem. Lett..

[37] Doronina S.O., Toki B.E., Torgov M.Y., Mendelsohn B.A., Cerveny C.G., Chace D.F., DeBlanc R.L., Gearing R.P., Bovee T.D., Siegall C.B. (2003). Development of potent monoclonal antibody auristatin conjugates for cancer therapy. Nat. Biotechnol..

[38] Jain N., Smith S.W., Ghone S., Tomczuk B. (2015). Current ADC Linker Chemistry. Pharm. Res..

[39] Dubowchik G.M., Firestone R.A., Padilla L., Willner D., Hofstead S.J., Mosure K., Knipe J.O., Lasch S.J., Trail P.A. (2002). Cathepsin B-labile dipeptide linkers for lysosomal release of doxorubicin from internalizing immunoconjugates: Model studies of enzymatic drug release and antigen-specific in vitro anticancer activity. Bioconjug. Chem..

[40] Caculitan N.G., Dela C., Chuh J., Ma Y., Zhang D., Kozak K.R., Liu Y., Pillow T.H., Sadowsky J., Cheung T.K. (2017). Cathepsin B Is Dispensable for Cellular Processing of Cathepsin B-Cleavable Antibody-Drug Conjugates. Cancer Res..

[41] Laguzza B.C., Nichols C.L., Briggs S.L., Cullinan G.J., Johnson D.A., Starling J.J., Baker A.L., Bumol T.F., Corvalan J.R. (1989). New antitumor monoclonal antibody-vinca conjugates LY203725 and related compounds: Design, preparation, and representative in vivo activity. J. Med. Chem..

[42] Govindan S.V., Cardillo T.M., Sharkey R.M., Tat F., Gold D.V., Goldenberg D.M. (2013). Milatuzumab-SN-38 conjugates for the treatment of CD74+ cancers. Mol. Cancer.

[43] Tranoy-Opalinski I., Legigan T., Barat R., Clarhaut J., Thomas M., Renoux B., Papot S. (2014). β-Glucuronidase-responsive prodrugs for selective cancer chemotherapy: An update. Eur. J. Med. Chem..

[44] Lyon R.P., Bovee T.D., Doronina S.O., Burke P.J., Hunter J.H., Neff-LaFord H.D., Jonas M., Anderson M.E., Setter J.R., Senter P.D. (2015). Reducing hydrophobicity of homogeneous antibody-drug conjugates improves pharmacokinetics and therapeutic index. Nat. Biotechnol..

[45] Kolodych S., Michel C., Delacroix S., Koniev O., Ehkirch A., Eberova J., Cianférani S., Renoux B., Krezel W., Poinot P. (2017). Development and evaluation of β-galactosidase-sensitive antibody-drug conjugates. Eur. J. Med. Chem..

[46] Lambert J.M., Chari R.V. (2014). Ado-trastuzumab Emtansine (T-DM1): An antibody-drug conjugate (ADC) for HER2-positive breast cancer. J. Med. Chem..

[47] Kovtun Y.V., Audette C.A., Ye Y., Xie H., Ruberti M.F., Phinney S.J., Leece B.A., Chittenden T., Blättler W.A., Goldmacher V.S. (2006). Antibody-drug conjugates designed toeradicate tumors with homogeneous and heterogeneous expression of the targetantigen. Cancer Res..

[48] Oflazoglu E., Stone I.J., Gordon K., Wood C.G., Repasky E.A., Grewal I.S., Law C.L., Gerber H.P. (2008). Potent anticarcinoma activity of the humanized anti-CD70 antibody h1F6 conjugated to the tubulin inhibitor auristatin via an uncleavable linker. Clin. Cancer Res..

[49] Polson A.G., Calemine-Fenaux J., Chan P., Chang W., Christensen E., Clark S., de Sauvage F.J., Eaton D., Elkins K., Elliott J.M. (2009). Antibody-drug conjugates for the treatment of non-Hodgkin’s lymphoma: Target and linker-drug selection. Cancer Res..

[50] Sau S., Alsaab H.O., Kashaw S.K., Tatiparti K., Iyer A.K. (2017). Advances in antibody-drugconjugates: A new era of targeted cancer therapy. Drug Discov. Today.

[51] Doronina S.O., Mendelsohn B.A., Bovee T.D., Cerveny C.G., Alley S.C., Meyer D.L., Oflazoglu E., Toki B.E., Sanderson R.J., Zabinski R.F. (2006). Enhanced activity of monomethylauristatin F through monoclonal antibody delivery: Effects of linker technology on efficacy and toxicity. Bioconjug. Chem..

[52] Lencer W.I., Blumberg R.S. (2005). A passionate kiss, then run: Exocytosis and recycling of IgG by FcRn. Trends Cell Biol..

[53] Chen H., Lin Z., Arnst K.E., Miller D.D., Li W. (2017). Tubulin inhibitor-based antibody-drug conjugates for cancer therapy. Molecules.

[54] Anderl J., Faulstich H., Hechler T., Kulke M. (2013). Antibody–Drug Conjugate Payloads. Methods Mol. Biol..

[55] Zakacs G., Paterson J.K., Ludwig J.A., Booth-Genthe C., Gottesman M.M. (2006). Targeting multidrug resistance in cancer. Nat. Rev. Drug Discov..

[56] Leung D., Wurst J.M., Liu T., Martinez R.M., Datta-Mannan A., Feng Y. (2020). Antibody Conjugates-Recent Advances and Future Innovations. Antibodies (Basel).

[57] Kaur G., Hollingshead M., Holbeck S., Schauer-Vukasinovic V., Camalier R.F., Domling A., Agarwal S. (2006). Biological evaluation of tubulysin A: A potential anticancer and antiangiogenic natural product. Biochem. J..

[58] Prota A.E., Bargsten K., Diaz J.F., Marsh M., Cuevas C., Liniger M., Steinmetz M.O. (2014). A new tubulin-binding site and pharmacophore for microtubule destabilizing anticancer drugs. Proc. Natl. Acad. Sci. USA.

[59] Dumontet C., Jordan M.A. (2010). Microtubule-binding agents: A dynamic field of cancer therapeutics. Nat. Rev. Drug Discov..

[60] Kastenhuber E.R., Lowe S.W. (2017). Putting p53 in Context. Cell.

[61] Yaghoubi S., Karimi M.H., Lotfinia M., Gharibi T., Mahi-Birjand M., Kavi E., Hosseini F., Sineh Sepehr K., Khatami M., Bagheri N. (2020). Potential drugs used in the antibody-drug conjugate (ADC) architecture for cancer therapy. J. Cell. Physiol..

[62] Antonow D., Thurston D.E. (2011). Synthesis of DNA-interactive pyrrolo[2,1-c][1,4]benzodiazepines (PBDs). Chem. Rev..

[63] Tietze L.F., Schmuck K. (2011). Prodrugs for targeted tumor therapies: Recent developments in ADEPT, GDEPT and PMT. Curr. Pharm. Des..

[64] Dokter W., Ubink R., van der Lee M., van der Vleuten M., van Achterberg T., Jacobs D., Loosveld E., van den Dobbelsteen D., Egging D., Mattaar E. (2014). Preclinical profile of theHER2-targeting ADC SYD983/SYD985: Introduction of a new duocarmycin-basedlinker-drug platform. Mol. Cancer.

[65] Rinnerthaler G., Gampenrieder S.P., Greil R. (2019). HER2 Directed Antibody-Drug-Conjugates beyond T-DM1 in Breast Cancer. Int. J. Mol. Sci..

[66] Gebleux R., Casi G. (2016). Antibody-drug conjugates: Current status and future perspectives. Pharm. Ther..

[67] Adams D.J., Dewhirst M.W., Flowers J.L., Gamcsik M.P., Colvin O.M., Manikumar G., Wani M.C., Wall M.E. (2000). Camptothecin analogues with enhanced antitumor activity at acidic pH. Cancer Chemother. Pharm..

[68] Starodub A.N., Ocean A.J., Shah M.A., Guarino M.J., Picozzi V.J., Vahdat L.T., Thomas S.S., Govindan S.V., Maliakal P.P., Wegener W.A. (2015). First-in-human trial of a novel anti-trop-2 antibody-SN-38 conjugate, sacituzumab govitecan, for the treatment of diverse metastatic solid tumors. Clin. Cancer Res..

[69] Yu Q., Ding J. (2015). Precision cancer medicine: Where to target?. Acta Pharmacol. Sin..

[70] Chowdari N.S., Pan C., Rao C., Langley D.R., Sivaprakasam P., Sufi B., Derwin D., Wang Y., Kwok E., Passmore D. (2019). Uncialamycin as a novel payload for antibody drug conjugate (ADC) based targeted cancer therapy. Bioorganic. Med. Chem. Lett..

[71] Kim E.G., Kim K.M. (2015). Strategies and advancement in antibody- drug conjugate optimization for targeted cancer therapeutics. Biomol. Ther. (Seoul).

[72] Hennessy E.J. (2016). Selective inhibitors of Bcl-2 and Bcl-xL: Balancing antitumor activity with on-target toxicity. Bioorganic. Med. Chem. Lett..

[73] Hallen H.E., Luo H., Scott-Craig J.S., Walton J.D. (2007). Gene family encoding the major toxins of lethal Amanita mushrooms. Proc. Natl. Acad. Sci. USA.

[74] Danielczyk A., Stahn R., Faulstich D., Löffler A., Märten A., Karsten U., Goletz S. (2006). PankoMab: A potent new generation anti-tumour MUC1 antibody. Cancer Immunol. Immunother. CII.

[75] Kaplan G., Mazor R., Lee F., Jang Y., Leshem Y., Pastan I. (2018). Improving the In Vivo Efficacy of an Anti-Tac (CD25) Immunotoxin by Pseudomonas Exotoxin A Domain II Engineering. Mol. Cancer Ther..

[76] Kaplan G., Lee F., Onda M., Kolyvas E., Bhardwaj G., Baker D., Pastan I. (2016). Protection of the Furin Cleavage Site in Low-Toxicity Immunotoxins Based on Pseudomonas Exotoxin A. Toxins.

[77] Tsuchikama K., An Z. (2018). Antibody-drug conjugates: Recent advances in conjugation and linker chemistries. Protein Cell.

[78] Liu H., May K. (2012). Disulfide bond structures of IgG molecules: Structural variations, chemical modifications and possible impacts to stability and biological function. mABs.

[79] Gébleux R., Wulhfard S., Casi G., Neri D. (2015). Antibody Format and Drug Release RateDetermine the Therapeutic Activity of Noninternalizing Antibody-Drug Conjugates. Mol. Cancer Ther..

[80] Anami Y., Xiong W., Gui X., Deng M., Zhang C.C., Zhang N., An Z., Tsuchikama K. (2017). Enzymatic conjugation using branched linkers for constructing homogeneous antibody-drug conjugates with high potency. Org. Biomol. Chem..

[81] Bruins J.J., Westphal A.H., Albada B., Wagner K., Bartels L., Spits H., van Berkel W.J.H., van Delft F.L. (2017). Inducible, Site-Specific Protein Labeling by Tyrosine Oxidation-Strain-Promoted (4 + 2) Cycloaddition. Bioconjug. Chem..

[82] Axup J.Y., Bajjuri K.M., Ritland M., Hutchins B.M., Kim C.H., Kazane S.A. (2012). Synthesis of site-specific antibody-drug conjugates using unnatural amino acids. Proc. Natl. Acad. Sci. USA.

[83] Tian F., Lu Y., Manibusan A., Sellers A., Tran H., Sun Y., Phuong T., Barnett R., Hehli B. (2014). A general approach to site-specific antibody drug conjugates. Proc. Natl. Acad. Sci. USA.

[84] Zimmerman E.S., Heibeck T.H., Gill A., Li X., Murray C.J., Madlansacay M.R., Tran C., Uter N.T., Yin G., Rivers P.J. (2014). Production of site-specific antibody-drug conjugates using optimized non-natural amino acids in a cell-free expression system. Bioconjug. Chem..

[85] Norsworthy K.J., Ko C.W., Lee J.E., Liu J., John C.S., Przepiorka D., Farrell A.T., Pazdur R. (2018). FDA Approval Summary: Mylotarg for Treatment of Patients with Relapsed orRefractory CD33-Positive Acute Myeloid Leukemia. Oncologist.

[86] Ricart A.D. (2011). Antibody-drug conjugates of calicheamicin derivative: Gemtuzumab ozogamicin and inotuzumab ozogamicin. Clin. Cancer Res..

[87] Tanimoto T., Tsubokura M., Mori J., Pietrek M., Ono S., Kami M. (2013). Differences in drugapproval processes of 3 regulatory agencies: A case study of gemtuzumabozogamicin. Invest. New Drugs.

[88] FDA (2017). FDA Approves Mylotarg for Treatment of Acute Myeloid leukemia [WWW]. https://www.fda.gov/newsevents/newsroom/pressannouncements/ucm574507.htm.

[89] FDA (2017). FDA Approves New Treatment for Adults with Relapsed or Refractory Acute Lymphoblastic Leukemia [WWW]. https://www.fda.gov/newsevents/newsroom/pressannouncements/ucm572131.htm.

[90] EMA Besponsa (2017). Inotuzumab ozogamicin [WWW]. http://www.ema.europa.eu/ema/index.jsp?curl=pages/medicines/human/medicines/004119/human_med_002109.jsp&mid=WC0b01ac058001d124.

[91] Lamb Y.N. (2017). Inotuzumab Ozogamicin: First Global Approval. Drugs.

[92] Moek K.L., de Groot D.J.A., de Vries E.G.E., Fehrmann R.S.N. (2017). The antibody-drug conjugate target landscape across a broad range of tumour types. Ann. Oncol..

[93] Dan N., Setua S., Kashyap V.K., Khan S., Jaggi M., Yallapu M.M., Chauhan S.C. (2018). Antibody-Drug Conjugates for Cancer Therapy: Chemistry to Clinical Implications. Pharmaceuticals (Basel).

[94] EMA Herceptin (2018). Trastuzumab [WWW]. http://www.ema.europa.eu/ema/index.jsp?curl=pages/medicines/human/medicines/000278/human_med_000818.jsp&mid=WC0b01ac058001d124.

[95] EMA Kadcyla (2018). Trastuzumab Emtansine [WWW]. http://www.ema.europa.eu/ema/index.jsp?curl=pages/medicines/human/medicines/002389/human_med_001712.jsp&mid=WC0b01ac058001d124.

[96] FDA Drug Approval Package (2013). Kadcyla (Ado-Trastuzumab Emtansine) Injection [WWW]. https://www.accessdata.fda.gov/drugsatfda_docs/nda/2013/125427Orig1s000TOC.cfm.

[97] PMDA Trastuzumab emtansine (2013). Review Report [WWW]. http://www.pmda.go.jp/files/000153735.pdf.

[98] Deeks E.D. (2019). Polatuzumab Vedotin: First Global Approval. Drugs.

[99] Dhillon S. (2018). Moxetumomab Pasudotox: First Global Approval. Drugs.

[100] Yoder N.C., Bai C., Tavares D., Widdison W.C., Whiteman K.R., Wilhelm A., Wilhelm S.D., McShea M.A., Maloney E.K., Ab O. (2019). A Case Study Comparing Heterogeneous Lysine- and Site-Specific Cysteine- Conjugated Maytansinoid Antibody-Drug Conjugates (ADCs) Illustrates the Benefits of Lysine Conjugation. Mol. Pharm..

[101] Goulet D.R., Atkins W.M. (2020). Considerations for the Design of Antibody-Based Therapeutics. J. Pharm. Sci..

[102] Deonarain M.P. (2018). Miniaturised ‘antibody’-drug conjugates for solid tumours?. Drug Discov. Today Technol..

[103] Cimini A., Mei S., Benedetti E., Laurenti G., Koutris I., Cinque B., Cifone M.G., Galzio R., Pitari G., Di Leandro L. (2012). Distinct cellular responses induced by saporin and a transferrin-saporinconjugate in two different human glioblastoma cell lines. J. Cell Physiol..

[104] Della Cristina P., Castagna M., Lombardi A., Barison E., Tagliabue G., Ceriotti A., Koutris I., Di Leandro L., Giansanti F., Vago R. (2015). Systematic comparison of single-chain Fvantibody-fusion toxin constructs containing Pseudomonas Exotoxin A or saporinproduced in different microbial expression systems. Microb. Cell Fact..

[105] Giansanti F., Di Leandro L., Koutris I., Pitari G., Fabbrini M.S., Lombardi A., Flavell D.J., Flavell S.U., Gianni S., Ippoliti R. (2010). Engineering a switchable toxin: Thepotential use of PDZ domains in the expression, targeting and activation ofmodified saporin variants. Protein Eng. Des. Sel..

[106] Giansanti F., Sabatini D., Pennacchio M.R., Scotti S., Angelucci F., Dhez A.C., Antonosante A., Cimini A., Giordano A., Ippoliti R. (2015). PDZ Domain in the Engineeringand Production of a Saporin Chimeric Toxin as a Tool for targeting Cancer Cells. J. Cell Biochem..

[107] Provenzano E.A., Posteri R., Giansanti F., Angelucci F., Flavell S.U., Flavell D.J., Fabbrini M.S., Porro D., Ippoliti R., Ceriotti A. (2016). Optimization of construct design and fermentation strategy for the production ofbioactive ATF-SAP, a saporin based anti-tumoral uPAR-targeted chimera. Microbcell Fact..

[108] Dhez A.C., Benedetti E., Antonosante A., Panella G., Ranieri B., Florio T.M., Cristiano L., Angelucci F., Giansanti F., Di Leandro L. (2018). Targeted therapy of human glioblastoma via delivery of a toxinthrough a peptide directed to cell surface nucleolin. J. Cell Physiol..

[109] Marques A.C., Costa P.J., Velho S., Amaral M.H. (2020). Functionalizing nanoparticles with cancer-targeting antibodies: A comparison of strategies. J. Control. Release.

[110] El Maghraby G.M., Arafa M.F. (2020). Liposomes for enhanced cellular uptake of anticancer agents. Curr. Drug Deliv..

[111] Sun H., Erdman W., Yuan Y., Mohamed M.A., Xie R., Gong S., Cheng C. (2020). Crosslinked polymer nanocapsules for therapeutic, diagnostic, and theranostic applications. Wiley Interdiscip. Rev. Nanomed. Nanobiotechnol..

[112] Jindal M., Nagpal M., Singh M., Aggarwal G., Dhingra G.A. (2020). Gold Nanoparticles- Boon in Cancer Theranostics. Curr. Pharm. Des..

[113] Ardini M., Huang J., Sánchez C.S., Mousavi M.Z., Caprettini V., Maccaferri N., Melle G., Bruno G., Pasquale L., Garoli D. (2018). Live Intracellular Biorthogonal Imaging by Surface Enhanced Raman Spectroscopy using Alkyne-Silver Nanoparticles Clusters. Sci. Rep..

[114] Wang H., Zheng M., Gao J., Wang J., Zhang Q., Fawcett J.P., He Y., Gu J. (2020). Uptake and release profiles of PEGylated liposomal doxorubicin nanoparticles: A comprehensive picture based on separate determination of encapsulated and total drug concentrations in tissues of tumor-bearing mice. Talanta.

[115] Johnston M.C., Scott C.J. (2018). Antibody conjugated nanoparticles as a novel form of antibody drug conjugate chemotherapy. Drug Discov. Today Technol..

[116] Coats S., Williams M., Kebble B., Dixit R., Tseng L., Yao N.S., Tice D.A., Soria J.C. (2019). Antibody-Drug Conjugates: Future Directions in Clinical and Translational Strategies to Improve the Therapeutic Index. Clin. Cancer Res..

[117] Drake P.M., Rabuka D. (2017). Recent Developments in ADC Technology: Preclinical Studies Signal Future Clinical Trends. Bio. Drugs.

